# AL Amyloidosis Complicated by Persistent Oral Bleeding

**DOI:** 10.1155/2015/981346

**Published:** 2015-05-07

**Authors:** Luiz Antonio Liarte Marconcini, Forrest Marc Stewart, Lisa Sonntag, Emily Stevens, Nicholas Burwick

**Affiliations:** ^1^University of North Dakota School of Medicine and Health Sciences, Grand Forks, ND 58202, USA; ^2^Fred Hutchinson Cancer Research Center, University of Washington School of Medicine, Seattle, WA 98109, USA; ^3^Fred Hutchinson Cancer Research Center, University of Washington School of Dentistry, Seattle, WA 98109, USA

## Abstract

A case of amyloid light chain (AL) amyloidosis is presented here with uncontrolled bleeding after a nonsurgical dental procedure, most likely multifactorial in nature, and consequently treated with a multidisciplinary approach.

## 1. Background

AL amyloidosis is identified as a disease process in which extracellular protein misfolding leads to fibril formation and deposits throughout the body. More than 20 different proteins have been identified [1, 2]. Disease progression is associated with end organ damage and related complications [3, 4].

AL amyloidosis is the most aggressive form of systemic amyloidosis [5]. The median survival without therapy is approximately one year [6]. This survival rate is noted to decrease with cardiac involvement [7].

Patients can present with macroglossia, purpura (characteristic periorbital distribution “raccoon eyes”), hepatomegaly, renal damage, restrictive cardiomyopathy, peripheral neuropathy, and* bleeding diathesis*. Approximately 10% of patients have coexisting multiple myeloma with associated symptoms (such as anemia and bone pain) [8].

The bleeding diathesis noted in patients with AL amyloidosis is proposed to have multiple pathogeneses. These range from deposition in end organs, amyloid affinity for clotting factors, and interference of fibrin formation [9]. As with our case, the oral cavity can be an area of deposition that eventually leads to diathesis complications. In a retrospective study by Stoopler et al., 2003, biopsies taken from 13 patients showed that 15 of the 17 samples had amyloid deposition in the subepithelial connective tissue [10].

## 2. Case

A 22-year-old woman presented to our hospital with uncontrolled gingival bleeding status after dental cleaning.

She had a history pertinent for multiple myeloma and AL amyloidosis complicated by cardiac and renal involvement.

Due to a need for dental work prior to autologous stem-cell transplantation with the purpose of avoiding oral complications associated with said transplant, the patient underwent a dental cleaning on the day prior to admission, which was complicated by gingival bleeding. She was evaluated at the clinic, given additional platelets, and directed to use gauze and direct pressure. She had transient improvement but eventually worsened with described bloody oozing of the gums.

Workup following admission was pertinent for normal levels of fibrinogen, PT, PTT, INR, vWF, factor 8, lupus inhibitor, and DIC panel. Hematocrit was low at 24 (from 32 the previous day) and platelet count was low at 81. Factor 10 was noted to be in the lower side of normal. Previous HIT testing one week before due to a concern for recent drop in platelet count in the outpatient setting was noted to be negative. This was thought to be secondary to the disease itself.

Over the first 2 days, RBCs and platelet infusions were given multiple times to maintain them at an adequate level for functioning.

Presumed coagulation factor deficiency was treated with vasopressin (DDAVP) followed by cryoprecipitate without improvement. Caution was used in regard to amount of treatment given due to the increased risk of thrombosis.

Due to the history of renal failure and consequent uremia, estrogen was given without improvement. However, following scheduled hemodialysis due to end stage renal disease secondary to the AL amyloidosis, bleeding still continued.

Since factor 10 was noted to be in the lower side of normal, vitamin K was also given to promote coagulation factor production in the liver.

**Figure 1:**
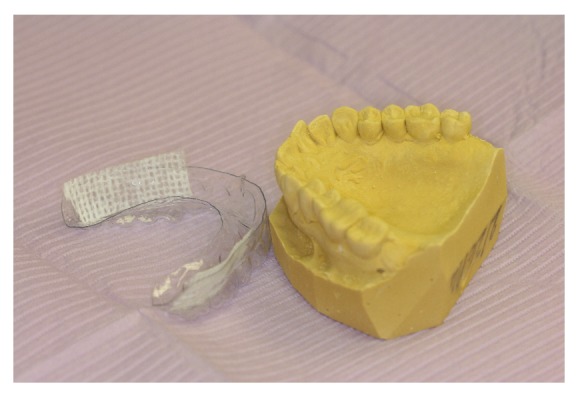
Dental appliances selectively lined with Surgicel according to areas with active hemorrhaging.

In the meantime, dental care management was initiated. Initially, aminocaproic acid Swish and Spit (an antifibrinolytic agent) was given with mild improvement. The following day, dental impressions were taken to fabricate maxillary and mandibular splint appliances (Figure 1). The dental appliances were selectively lined with Surgicel according to areas with active hemorrhaging prior to delivery. This was noted to have the best results in terms of decreasing the amount of hemorrhage. The patient found this treatment to be comfortable and convenient. She was able to continue her liquid and soft diet with the appliances in place.

Over the following days, the bleeding was eventually controlled with the combination of mouth packing, vitamin K, and desmopressin.

As is likely the case for most people affected with this disease, the etiology of bleeding diathesis can be multifactorial. In this case, the bleeding was likely due to a decrease in factor activity, platelet dysfunction, and local amyloid deposits. In addition, a component of heparin used during hemodialysis must also be considered in the equation.

## 3. Discussion

The major mechanism of bleeding in AL amyloidosis is thought to be due to amyloid deposit infiltration of the vasculature and musculature, leading to amyloid angiopathy, frailty, impaired vasoconstriction, and consequent tears and hemorrhage [11, 12]. In addition, coagulation factor deficiencies are noted in patients with AL amyloidosis [13, 14]. This is most likely due to the preferential involvement of the liver and spleen associated with the condition. This effect can be augmented in renal dysfunction. Renal involvement can lead to renal failure, uremia, and consequent platelet dysfunction.

There are no specific guidelines for treatment of AL amyloidosis related hemorrhage and control can be difficult. Therapy is dependent on the most likely etiology of the hemorrhage.

Coagulation factor deficiencies are managed with vitamin K, fresh frozen plasma, and cryoprecipitate. Desmopressin can be used with vWF and factor VIII deficiency. If bleeding is not controlled, specific clotting factors can be used. As expected, there is an associated increased risk of thrombosis with these treatments.

Platelet dysfunction due to renal involvement can be managed with estrogen but is also associated with an increased risk of thrombosis.

In our patient, the addition of splint appliances with Surgicel and consequent consistent pressure at the site of diathesis was noted to be helpful and arguably the main factor in hemorrhage control.

There is no definitive therapy for amyloid deposition related diathesis. Long-term management involves treating systemic amyloidosis itself. An aggressive approach involves high dose chemotherapy followed by autologous stem-cell transplantation. Studies have shown complete remission with improved survival rates in patients who undergo such treatment [7, 15]. However, caution must be used, as there is significant morbidity and mortality associated with this treatment, especially in those with cardiac involvement [7]. New therapy under investigation includes the immunomodulatory and antiangiogenic lenalidomide. In a recent study by Nagano et al., a patient with intractable melena in the setting of intestinal myeloma associated AL amyloidosis was noted to improve with lenalidomide as the curative treatment [16].

## 4. Conclusion

Unfortunately, as was the case with our patient, diathesis in AL amyloidosis can be multifactorial in etiology and difficult to treat [14]. Available therapy is mainly supportive. Here we presented a case where a splint appliance with its consistent pressure can be helpful to control hemorrhage.
